# Supplementary material for: NOTCH-mediated non-cell autonomous regulation of chromatin structure during senescence
Source: Nat Commun. 2018 May 9;9:1840. doi: 10.1038/s41467-018-04283-9 (PMC5943456; doi:10.1038/s41467-018-04283-9)
Supplement: Supplementary file 1 — Supplementary Information [file 41467_2018_4283_MOESM1_ESM.pdf]

Supplementary Information

NOTCH-mediated non-cell autonomous regulation of chromatin structure during senescence

Parry et al., 2018

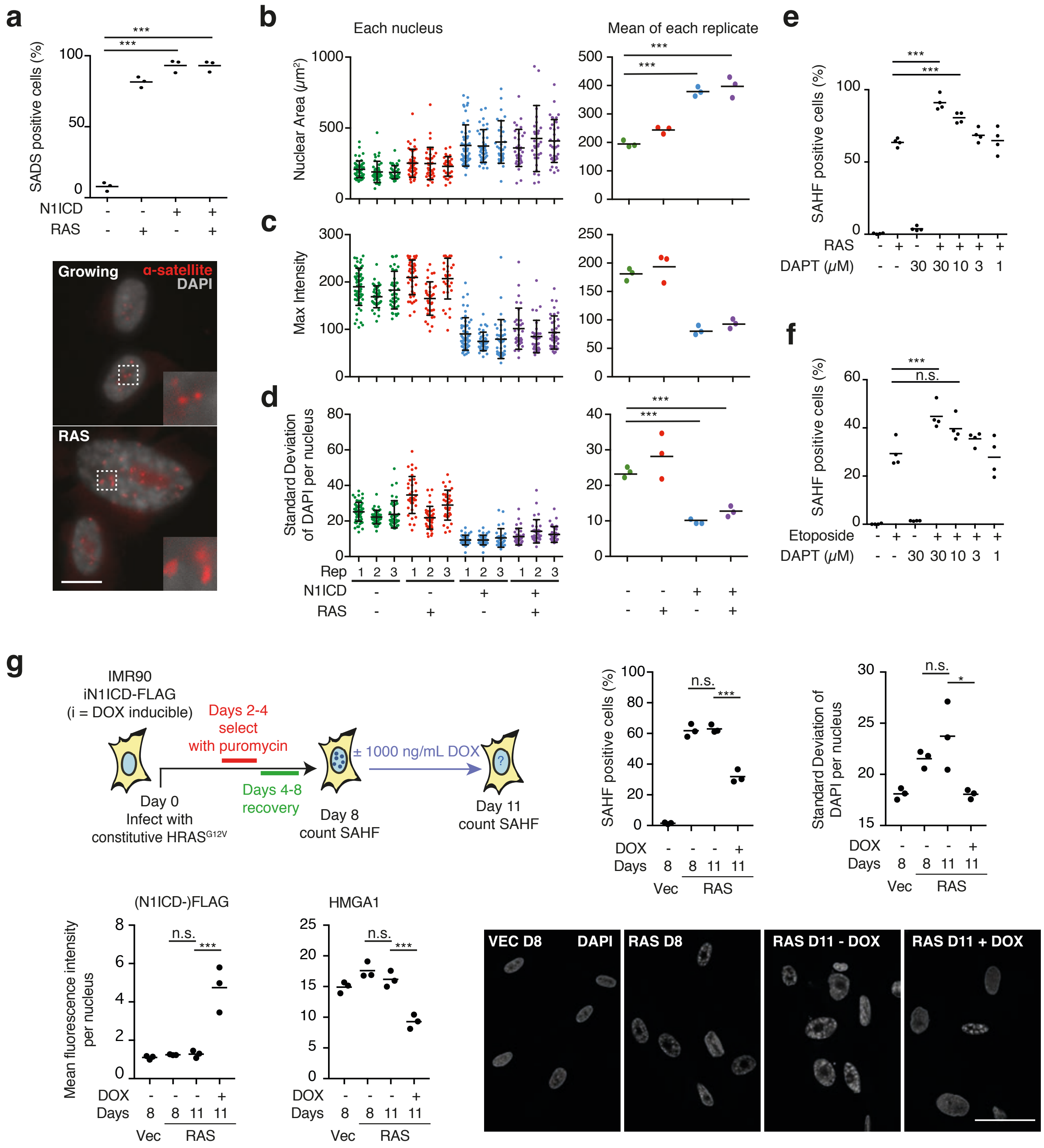

Supplementary Figure 1. NOTCH1 signalling has a chromatin ‘smoothening’ effect that strongly blocks SAHF formation **(a-d)** IMR90 ER:HRAS^{G12V} cells infected with control vector or N1ICD-FLAG ± 100 nM 4OHT. **(a)** Percentage of SADS positive cells by DNA-FISH (top) and representative images (bottom). Scale bar = 10 μm. n= 3 biological replicates. Inset is a zoomed image of the indicated region. **(b-d)** Quantification of nuclear area (b), maximum pixel intensity (c) and standard deviation (d) of signal from at least 30 DAPI stained nuclei per biological replicate for the conditions indicated. Data for individual nuclei (left, mean ± s.d is plotted). Means of each replicate (right) are also included for a direct comparison (also presented in Fig. 1c). n = 3 biologically independent replicates. **(e, f)** Quantification of SAHF positive cells in IMR90 ER:HRAS^{G12V} cells ± 100 nM 4OHT for 6 days **(e)** or IMR90 cells treated with 100 μM etoposide for 2 days, followed by 5 days drug free **(f)** together with different concentrations of DAPT. n = 4 biological replicates. **(g)** IMR90 cells with doxycycline (DOX)-inducible N1ICD were infected with constitutive HRASG12V until senescence was fully established (day 8 post selection). Cells were treated with ± 1000 ng/mL DOX for a further 3 days (day 11 post selection) and SAHF positive cells were counted. Vec = control vector; RAS = constitutive HRASG12V. Standard deviation of signal from at least 30 DAPI stained nuclei per replicate of cells described in the diagram. Average intensity of (N1ICD-)FLAG and HMGA1 IF staining per nucleus for the indicated cells. Representative images of the indicated cells are shown. Scale = 50 μm. (a-g) Statistical significance calculated using one-way ANOVA with Tukey’s correction for multiple comparisons. *$p \leq 0.05$, **$p \leq 0.01$, ***$p \leq 0.001$. n.s. = not signif-

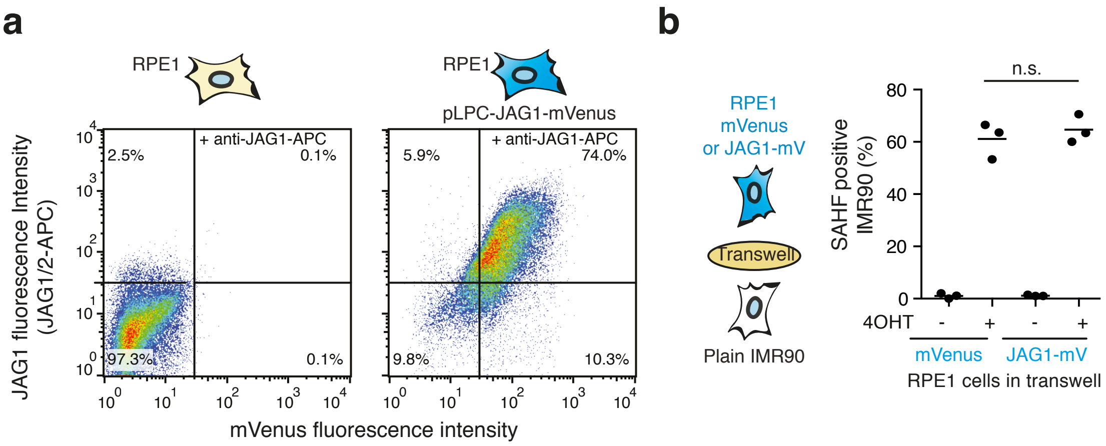

Supplementary Figure 2. Ectopic JAG1 expressing RPE1 cells require cell-cell contact to repress SAHFs in RIS cells **(a)** RPE1 JAGGED1-mVenus cells analysed for cell surface expression of JAG1/2 by flow cytometry. **(b)** Quantification of SAHF positive IMR90 ER:HRAS^{G12V} cells cultured in a transwell dish with the indicated RPE1 cells for 6 days ± 100 nM 4OHT. n= 3 biological replicates. Statistical significance calculated using one-way ANOVA with Tukey's correction for multiple comparisons. n.s. = not significant.

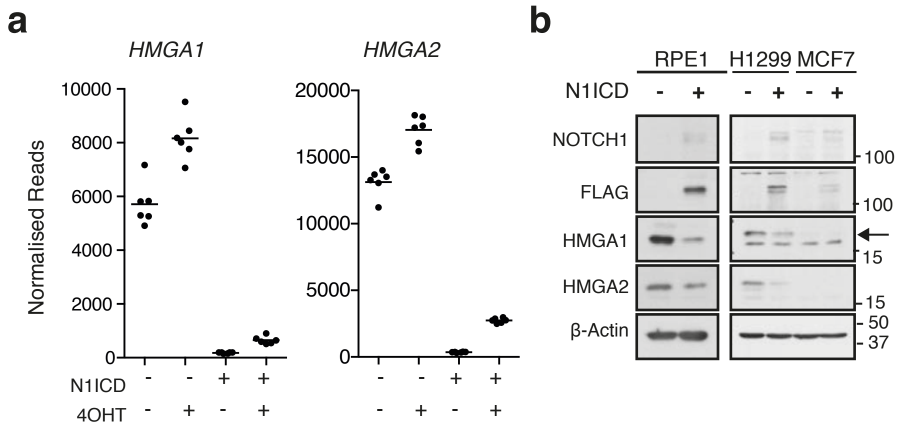

Supplementary Figure 3. NOTCH signalling represses HMGAs **(a)** Re-analysis of previously published RNA-seq data (GSE72404) for the cell conditions and genes indicated. **(b)** Immunoblotting of RPE1, H1299 and MCF7 cells expressing N1ICD-FLAG for the proteins indicated.

a

Sample	Replicate	Reads	RiP%
Growing	1	3615275	22.1
	2	4793280	15.3
	3	6333644	11.6
	4	5764813	13.3
RIS	1	4762672	32.1
	2	5082671	25.5
	3	4702537	25.7
NIS	1	3411360	5.73
	2	2302229	42.8
	3	2350015	43.6
N+RIS	1	3182849	8.48
	2	2662038	34.5
	3	2524354	34.7
RIS+shHMGA1	1	1363039	41.9
	2	1171948	40.5
	3	1252096	42.0
RIS+MCF7	1	3950321	34.4
	2	4834589	35.5
RIS+A549	1	3390583	41.8
	2	3968744	36.0
RIS+Hep3B	1	4944649	17.8
	2	4043354	19.3

b

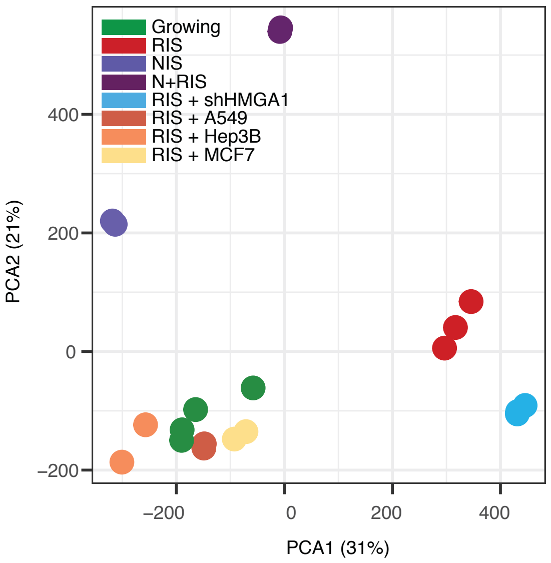

c

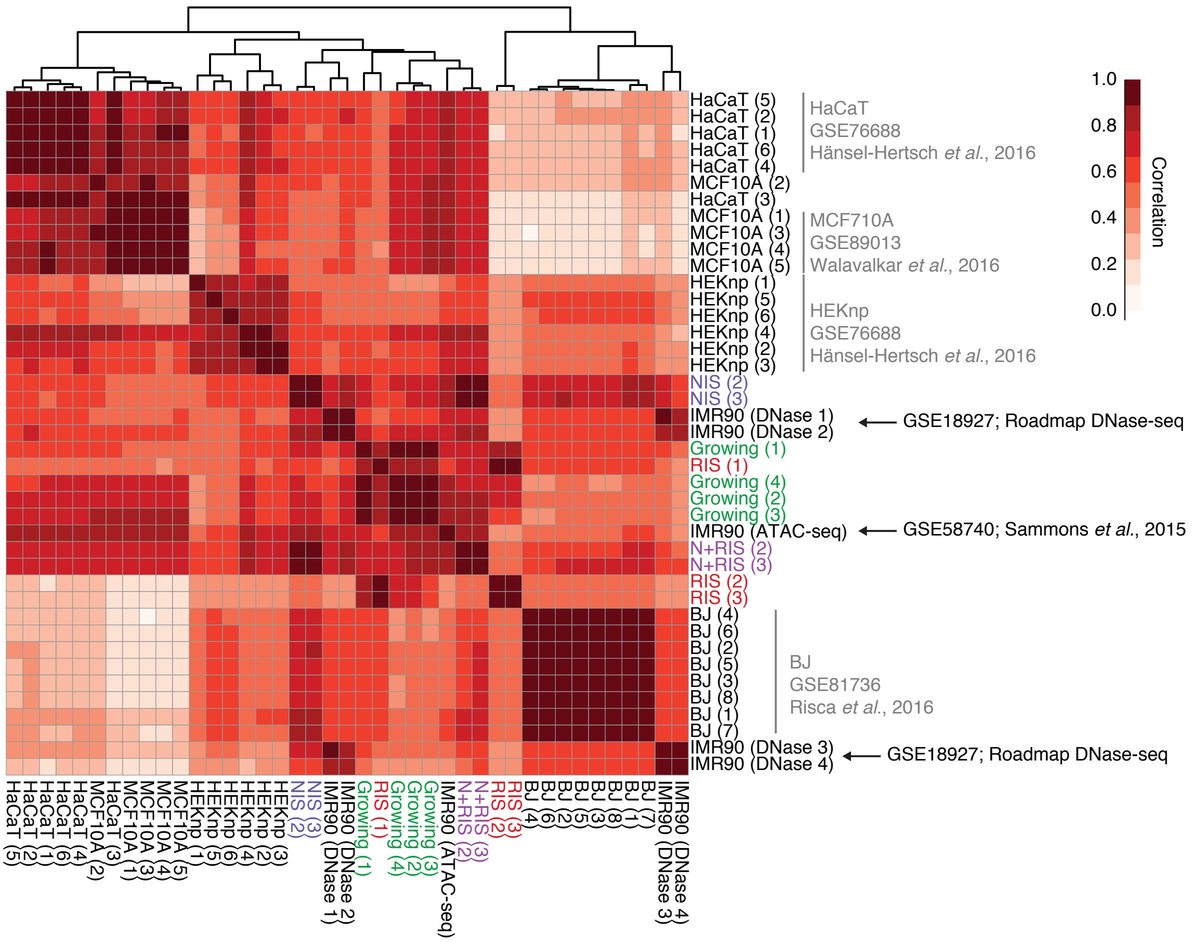

Supplementary Figure 4. ATAC-seq data is of high quality **(a)** Number of valid reads and percentage of reads in peaks (RiP%) for the ATAC-seq samples indicated. **(b)** Unbiased PCA analysis of normalised ATAC-seq samples. **(c)** Unbiased clustering analysis of ATAC-seq samples generated in the current study and publically available ATAC-seq and DNase-Seq data (as indicated).

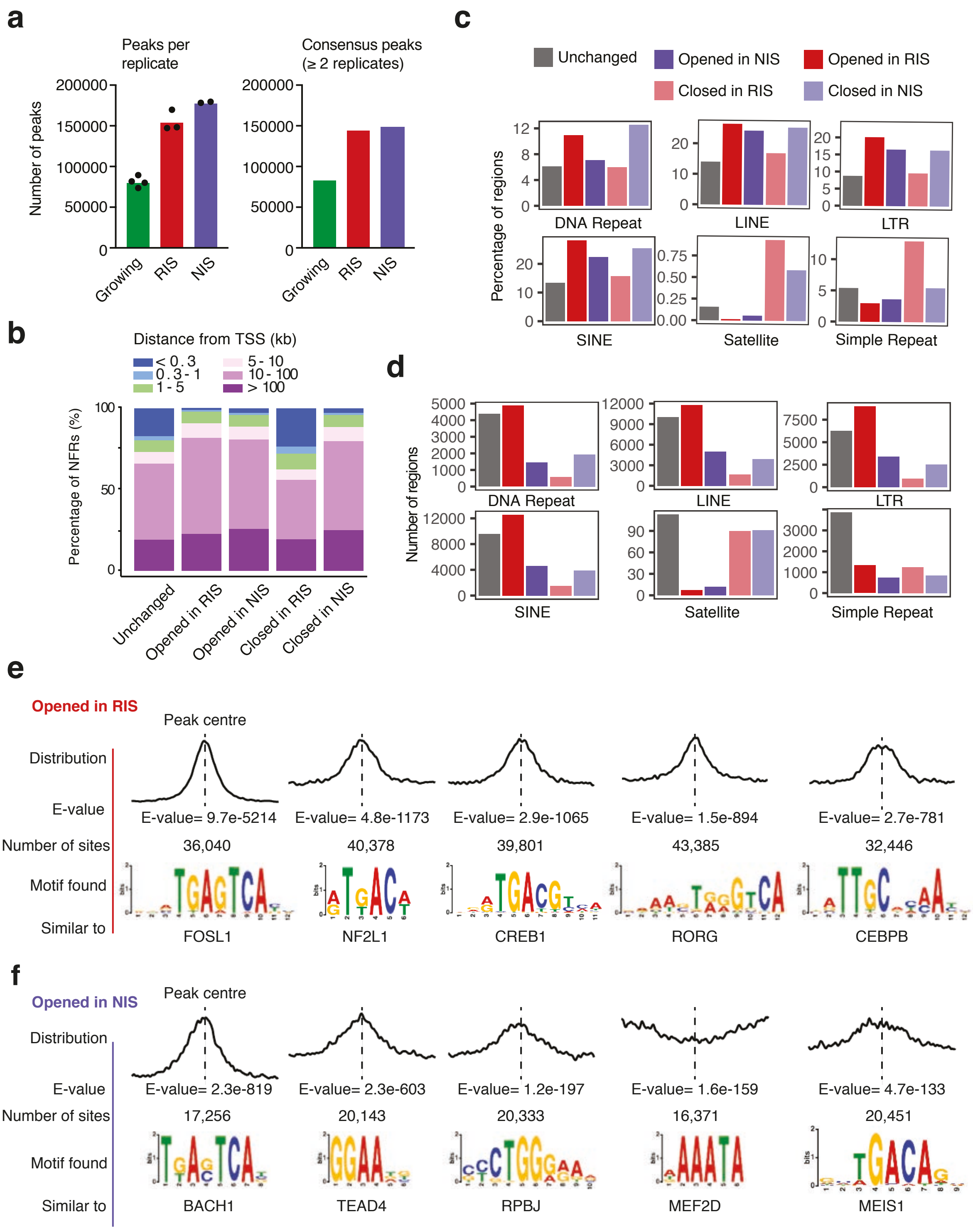

Supplementary Figure 5. Novel accessible regions in RIS and NIS are gene distal and enriched within repeat elements. **(a)** Number of ATAC-seq peaks detected in individual replicates from Growing, RIS and NIS cells (left) and consensus peaks (right, detected in at least 2 replicates) **(b)** Percentage of accessible regions in the categories indicated within indicated distances of a transcriptional start sites (TSS). **(c, d)** Percentage **(c)** and number **(d)** of accessible regions in the categories indicated annotated to repeat types. **(e, f)** Motif enrichment analysis of accessible regions in the categories indicated. 'Opened' = more accessible than in growing; 'Closed' = less accessible than in growing.

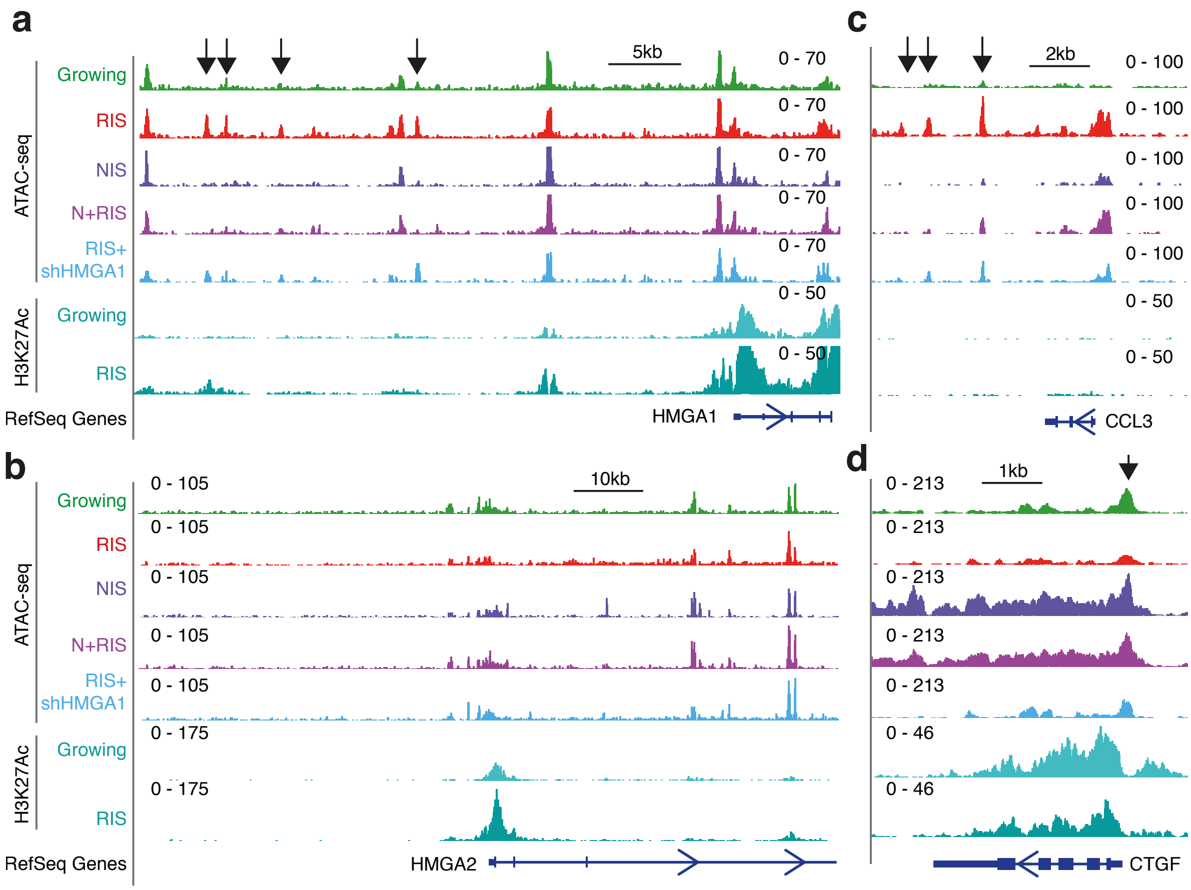

Supplementary Figure 6. Novel accessible regions in RIS are repressed by N1ICD. **(a-d)** Genome browser images of ATAC-seq and ChIP-seq for H3K27ac around genes of interest. Arrows indicate regions where accessibility is significantly altered in at least one of the conditions. (b) Accessible regions flanked by H3K27ac (putative enhancers) form upstream of HMGA1 in RIS and accessibility is repressed by N1ICD.

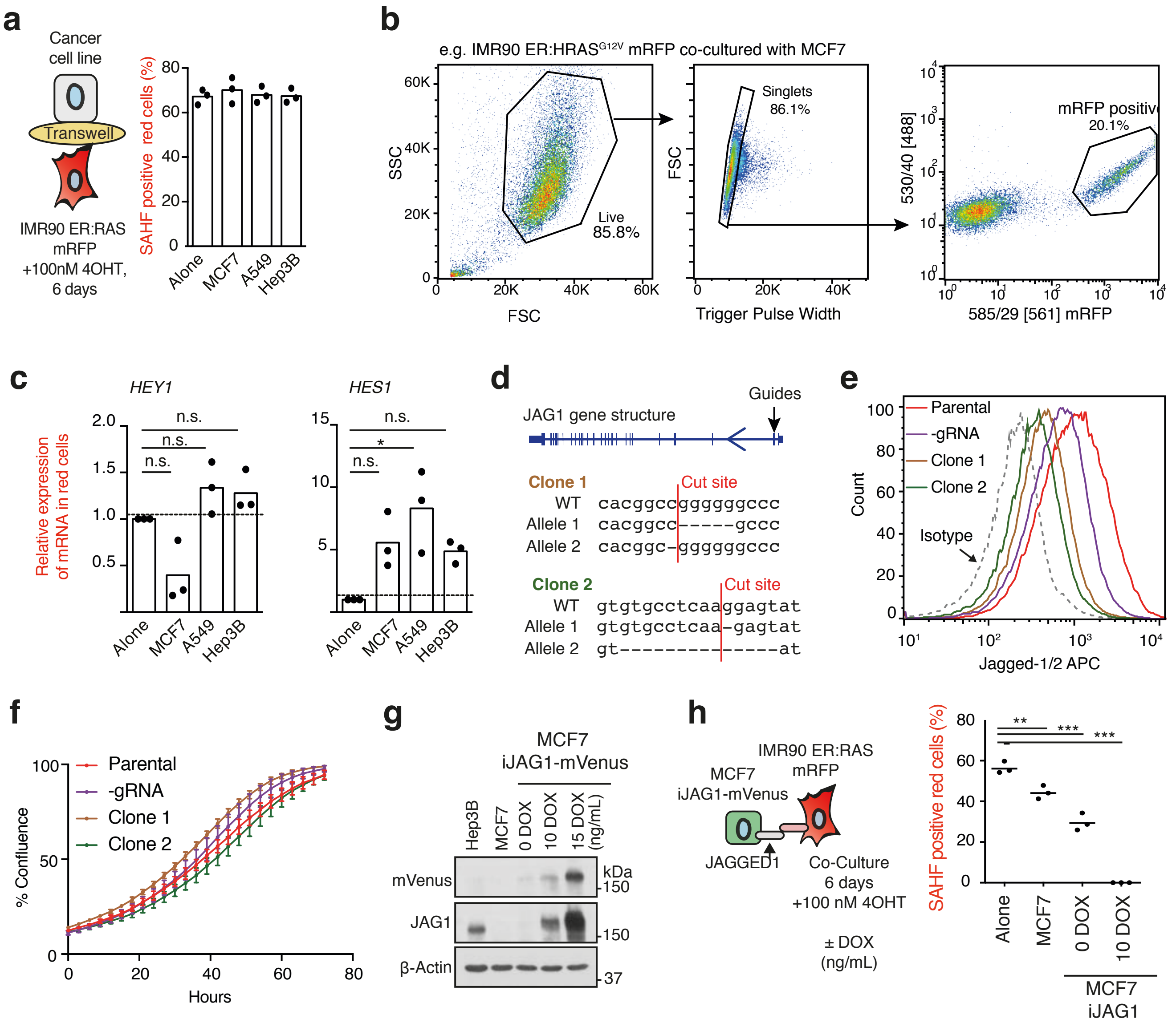

Supplementary Figure 7. Tumour cells can repress SAHF formation and chromatin opening in adjacent RIS fibroblasts. **(a)** Quantification of SAHF positive IMR90 ER:HRAS^{G12V} cells cultured in a transwell dish with the indicated tumour cell lines cells for 6 days + 100 nM 4OHT. n = 3 biological replicates. **(b)** Example of flow-sorting strategy for the isolation of mRFP positive cells from co-cultures between tumour cell lines and IMR90 ER:HRAS^{G12V} cells expressing mRFP. **(c)** qRT-PCR of mRNA isolated from flow sorted IMR90 ER:HRAS^{G12V} mRFP1 cells cultured with tumour cell lines + 100 nM 4OHT for 6 days relative to cells cultured alone (as described in Fig. 6d). n = 3 biological replicates. **(d)** Strategy for CRISPR editing of JAG1 in A549 cells and the sequence of the alleles in clone 1 and clone 2. **(e)** Cell surface expression of JAG-1/2 ligand as detected by flow cytometry. **(f)** Growth rate of the indicated A549 cells. n = 2 biological replicates each of 3 technical replicates. **(g)** Immunoblotting of MCF7 cells with doxycycline (DOX)-inducible JAG1-mVenus (iJAG1-mVenus) for JAG1 and mVenus treated with different concentrations of DOX for 3 days. **(h)** SAHF counting in IMR90 ER:HRAS^{G12V} cells expressing mRFP co-cultured with MCF7 expressing DOX-inducible JAG1-mVenus ± 100 nM 4OHT and ± 10 ng/mL DOX for 6 days. n = 3 biological replicates. **(b, c, h)** Statistical significance calculated using one-way ANOVA with Tukey's correction for multiple comparisons; *p ≤ 0.05, **p ≤ 0.01, ***p ≤ 0.001. n.s. = not significant.

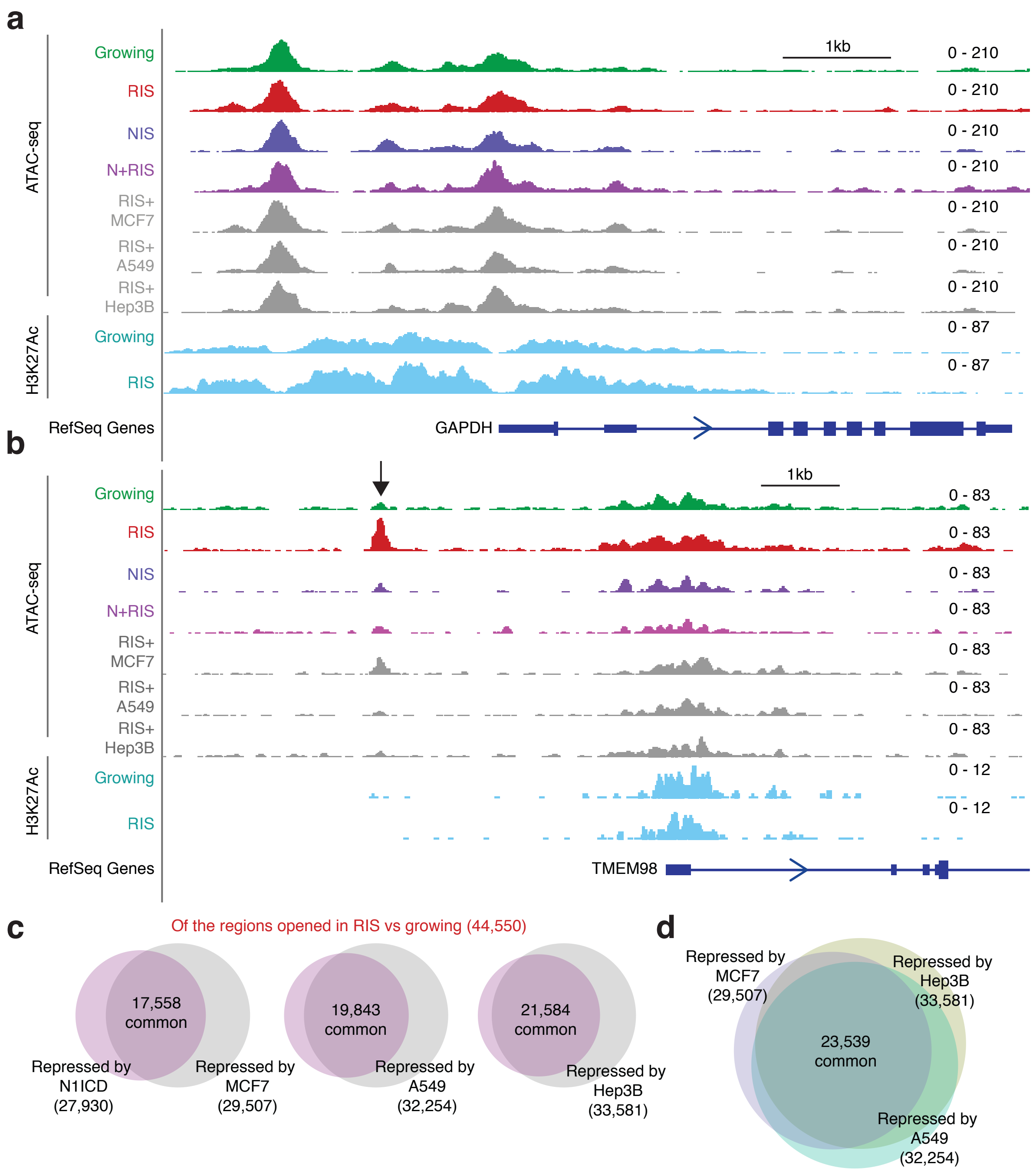

Supplementary Figure 8. Tumour cells can repress SAHFs and chromatin opening in adjacent RIS fibroblasts. **(a,b)** Genome browser images showing ATAC-seq data and ChIP-seq for H3K27ac in the conditions indicated. Note that '+MCF7', '+A549' and '+Hep3B' denotes that RIS cells have previously been cocultured with these cells for 6 days prior to flow sorting (as in Fig. 6d). The arrow indicates a region that becomes accessible in RIS and is significantly repressed by co-culture with A549 and Hep3B cells. **(c,d)** Venn diagrams showing literal overlap between accessible regions in the categories indicated. Novel accessible regions in RIS relative to growing (identified in Fig. 4c) that are repressed by tumour cell lines overlap well with the regions that are repressed by N1ICD (N+RIS vs RIS) **(c)** and with each other **(d)**.

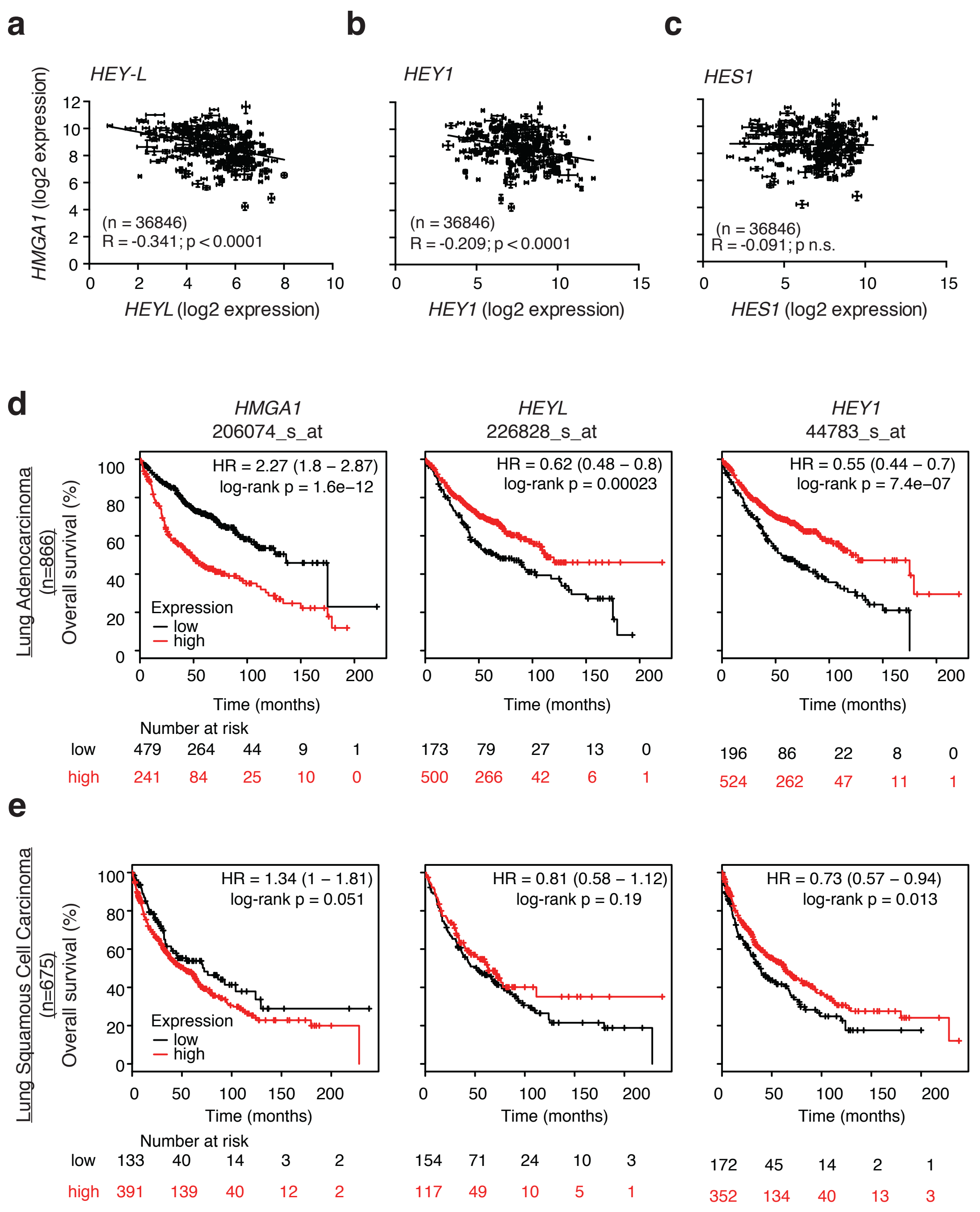

Supplementary Figure 9. *HMGA1* gene expression anti-correlates with canonical NOTCH target genes and is prognostic in cancer. **(a-c)** The R2 database (http://r2.amc.nl) was used to compare the microarray determined mRNA expression of *HMGA1* and *HEYL*, *HEY1* or *HES1* across all tissues and disease types present within this dataset. **(d, e)** Kaplan-Meier plots of overall survival of 866 patients with lung adenocarcinoma **(d)** and 675 patients with lung squamous cell carcinoma **(e)** stratified based on microarray determined mRNA expression of *HMGA1* (left panels), *HEYL* (middle panels) and *HEY1* (right panels). n.s. = not significant.

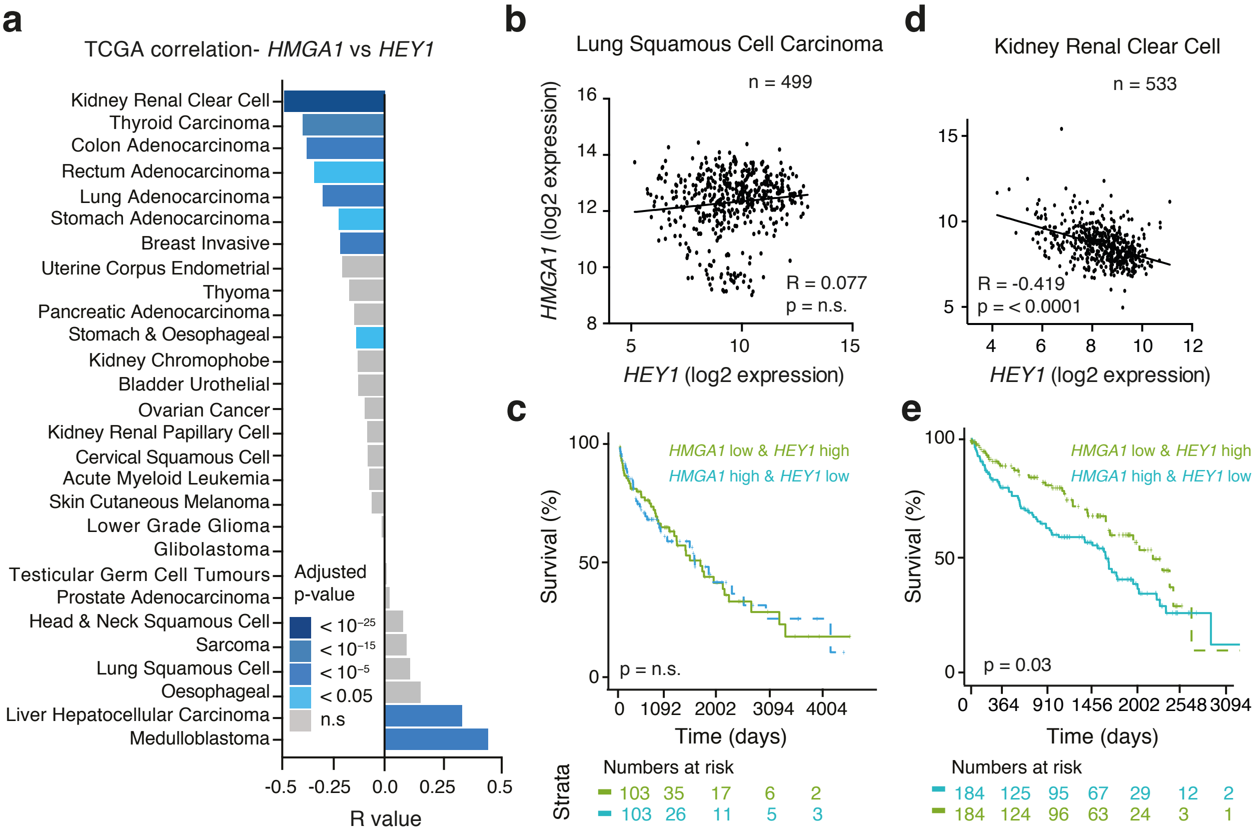

Supplementary Figure 10. (a) Pan-cancer analysis of the TCGA database. Correlation between HMGA1 and HEY1 is plotted in the indicated tumour types. Colours represent Bonferroni adjusted p-values on Pearson's correlation p-values. n.s. = not significant. **(b)** Log2 expression of HMGA1 against HEY1 in Lung Squamous Cell Carcinoma (LSCC). **(c)** Kaplan-Meier plot showing survival of LSCC patients stratified by HMGA1 and HEY1 gene expression. **(d)** Log2 expression of HMGA1 against HEY1 in Kidney Renal Clear Cell cancer (KIRC). **(e)** Kaplan-Meier plot showing survival of KIRC patients stratified by HMGA1 and HEY1 gene expression

Fig. 2b

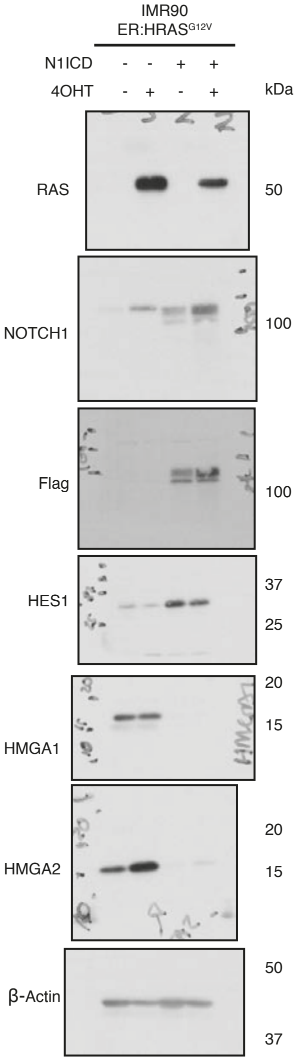

Fig. 2d

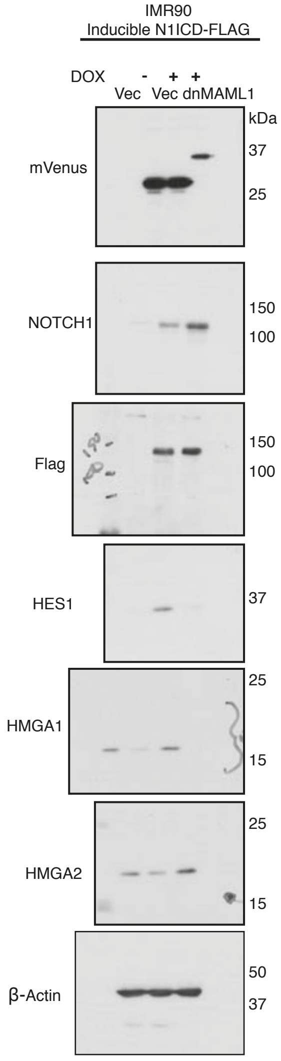

Fig. 6a

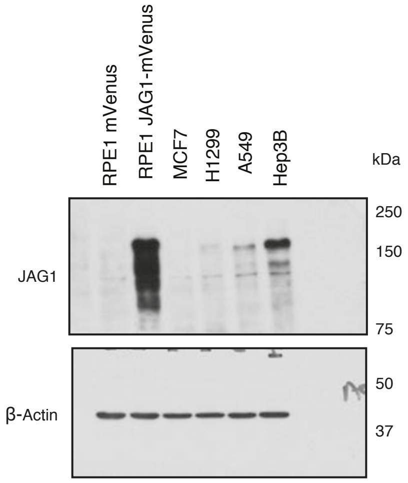

Fig. 6e

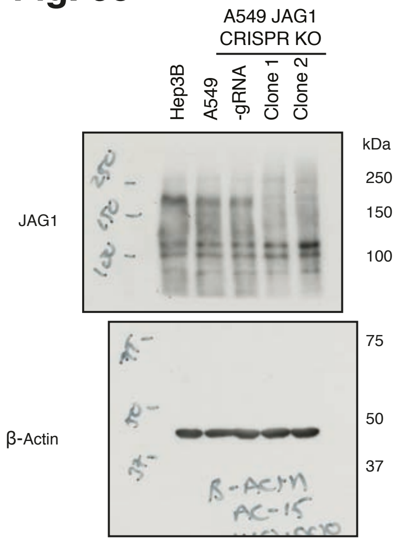

Supplementary Figure 11. Uncropped immunoblots used in the main figures for the proteins indicated.
