# Supplementary material for: NOTCH-mediated non-cell autonomous regulation of chromatin structure during senescence
Source: Nat Commun. 2018 May 9;9:1840. doi: 10.1038/s41467-018-04283-9 (PMC5943456; doi:10.1038/s41467-018-04283-9)
Supplement: Supplementary file 3 — Description of Additional Supplementary Files [file 41467_2018_4283_MOESM3_ESM.pdf]

**Description of Additional Supplementary Files**

**File Name: Supplementary Data 1**
**Description:** Annotation of accessible regions. Regions of chromatin that become significantly more or less accessible in RIS or NIS cells relative to growing cells were annotated using Homer. For each region, the distance to the closest transcriptional start site, the name of the closest gene and the GC percentage of the accessible region is given.
